# Modulation of Trehalose Dimycolate and Immune System by Rv0774c Protein Enhanced the Intracellular Survival of *Mycobacterium smegmatis* in Human Macrophages Cell Line

**DOI:** 10.3389/fcimb.2017.00289

**Published:** 2017-06-30

**Authors:** Arbind Kumar, Varinder Saini, Anjani Kumar, Jasbinder Kaur, Jagdeep Kaur

**Affiliations:** ^1^Department of Biotechnology, Panjab UniversityChandigarh, India; ^2^Department of Pulmonary Medicine, Government Medical College and HospitalChandigarh, India; ^3^Department of Biochemistry, Government Medical College and HospitalChandigarh, India

**Keywords:** *Mycobacterium* tuberculosis, Rv0774c, *Mycobacterium smegmatis*, trehalose dimycolate, intracellular survival, immune modulation

## Abstract

*Mycobacterium tuberculosis* Rv0774c protein was reported previously to express under stress conditions. Therefore, *Rv0774c* gene was cloned and expressed in *Mycobacterium smegmatis*, a surrogate host, to determine its role in bacterial persistence and immune modulation in natural environment. The bacterial colonies expressing *Rv0774c* (*Ms_rv0774c*) were larger, smoother, more moist, and flatter than the control ones (Ms_ve). Enhanced survival of Ms_rv0774c after treatment with streptomycin was observed when compared with control. The cell envelope of Ms_rv0774c was demonstrated to have more trehalose di-mycolate (TDM) and lesser amount of mycolylmannosylphosphorylheptaprenol (Myc-PL) in comparison to control. Higher intracellular survival rate was observed for *Ms_rv0774c* as compared to *Ms_ve* in the THP-1 cells. This could be correlated to the reduction in the levels of reactive NO and *iNOS* expression. Infection of macrophages with *Ms_rv0774c* resulted in significantly increased expression of TLR2 receptor and IL-10 cytokines. However, it lowered the production of pro-inflammatory cytokines such as IL-12, TNF-α, IFN-γ, and MCP-1 in *Ms_rv0774c* infected macrophages in comparison to the control and could be associated with decreased phosphorylation of p38 MAPK. Though, predicted with high antigenicity index bioinformatically, extracellular in nature and accessible to host milieu, Rv0774c was not able to generate humoral response in patient samples. Overall, the present findings indicated that Rv0774c altered the morphology and streptomycin sensitivity by altering the lipid composition of *M. smegmatis* as well as modulated the immune response in favor of bacterial persistence.

## Introduction

Tuberculosis (TB), a potentially deadly disease, is a bacterial infection caused by *Mycobacterium tuberculosis*. Despite the widely accepted BCG vaccines and various anti-TB drugs against this bacterium, it is still a major health concern for human beings. Indiscriminate or improper use of anti-TB drugs has led to the emergence of drug-resistant strains of *M. tuberculosis*; this has worsened the situation in recent years. Therefore, there is a need to identify new promising drug targets to treat drug-resistant TB cases, and it completely depends upon the understanding of the pathogenesis and intracellular survival of *M. tuberculosis*. The whole genome sequence of the *M. tuberculosis* H37Rv has been deciphered (Cole et al., 1998). Approximately, 4,000 gene products have been predicted to be involved in its metabolism and pathogenesis. Out of these, 40–50% genes have been identified as hypothetical. However, these might have some potential role in the biology of the bacterium.

As previously reported, few hypothetical mycobacterium proteins actively participated in the complex physiology of *M. tuberculosis* (Garg et al., 2015; Kumar et al., 2017). Nevertheless, the role of several other hypothetical proteins or proteins with unassigned function in the virulence and pathogenesis of this bacterium still remains unexplored/ poorly defined. PE/PPE protein family is exclusively found in the genome of *Mycobacterium* sp.; however, a majority of these proteins have not been assigned any function. Recent studies have revealed that these proteins actively participate in the host-pathogen interaction and modulate the host immune response in order to protect mycobacteria during infection and intracellular survival (Sampson, 2011). PE-PPE protein family contains conserved 110 and 180 Pro(P)-Pro(P)-Glu(E) amino acid residues at N-terminal and a significant variable C-terminal region (Cole and Barrell, 1998; Bottai and Brosch, 2009). It has been observed that the expression of *Rv1169c* (PE11) in *M. smegmatis* imparts significant resistance against the currently used anti-TB drugs and under various stress conditions by altering the lipid contents at mycobacterial cell surface. Additionally, Rv1169c has been observed to promote the survival of *M. smegmatis* in infected macrophages by altering the levels of IL-10, IL-4, and TNF-α cytokines (Singh et al., 2016). Overall, many others PPE/PE proteins were found to inhibit host's protective response and subsequently, increase susceptibility to mycobacterial infection (Basu et al., 2007; Nair et al., 2009; Tiwari et al., 2012; Khubaib et al., 2016; Bhat et al., 2017). The immunosuppressive nature of these proteins ignites their future aspect as probable drug targets against drug-resistant strains of *M. tuberculosis*.

In our previous study, we have reported that Rv0774c has sequence similarity with other known lipases, containing characteristic lipase/esterase signature motifs GXSXG, HG, and conserved catalytic residues such as Ser-185, Asp-255, and His-281 and degraded the short chain & mid chain pNP-esters (Kumar et al., 2016). Subsequently, Rv0774c protein was reported to be paralog of PE/PPE protein and expressed in iron limiting condition. It was identified in culture filtrate of *M. tuberculosis* H37Ra. The purified recombinant protein modulated the LPS mediated immune response (Kumar et al., 2017). These observations pointed toward its role in supporting intracellular survival of bacteria. Despite this, Rv0774c has shown similarity with the predominant secretary Ag85 complex proteins (mycolyl transferase) of *M. tuberculosis* which also possesses canonical α/β hydrolase fold; a catalytic triad of Ser, Glu, and His (Takayama et al., 2005). The unique characteristic of these proteins were being used to modify the traditional BCG vaccine to increase its efficacy. Additionally, protein-protein interaction analysis through STRING also revealed the co-occurrence of mmpL proteins of *M. tuberculosis* with Rv0774c (Supplementary Figure 1). These proteins were reported to be associated with mycolic acid metabolism and essential for *M. tuberculosis* viability, cell wall structure, and intrinsic resistance to anti-TB drugs (Barkan et al., 2009). Therefore, attempts were made in the present investigation to evaluate the effect of Rv0774c on growth, morphology, cell wall lipid profile, drug tolerance and intracellular survival of the bacterium in a near natural system. For the studies involving genes/proteins from pathogenic mycobacterium, *M. smegmatis* strain mc^2^ 155 has been used as a surrogate host (Etienne et al., 2005). In addition, *M. smegmatis* system can produce protein in native form and has nearly similar machinery for secreting the proteins to the growth medium as well as for the post translation modifications of protein, if there are any. In this study, we cloned and expressed *Rv0774c* gene from *M. tuberculosis* H37Rv into *M. smegmatis* strain mc^2^ 155 (*Ms_rv0774c*). Expression of Rv0774c alters the lipid profile of surface of *M. smegmatis*, which may resist the susceptibility against streptomycin. In addition to this, Rv0774c has shown effect on growth parameters and intracellular survival under *ex vivo* conditions, as well as on immune modulation in human macrophages cell line (THP-1).

## Materials and methods

### Ethical clearance and bacterial strains

Serum samples of TB patients were collected from Government Medical College and Hospital (GMCH), Sec-32, Chandigarh, India. The collection of human serum samples was approved by the Institute Ethics Committee of GMCH, Chandigarh, India and written consent was obtained from all participants. All experiments were conducted in accordance with the Ethical Guidelines for Biomedical Research on Human Subjects, Indian Council of Medical Research and Government of India.

*E. coli* DH5α and *Mycobacterium smegmatis* mc^2^155 were procured from IMTECH-MTCC, Chandigarh. The *E. coli- Mycobacterium* shuttle vector (pVV16) was a kind gift from Dr. Stephen Cannan, France. The middle brook 7H9 broth, glycerol, and tween-80 were purchased from Hi Media, India. The supplement OADC was purchased from BD bioscience (India). RPMI media and Fetal bovine serum (FBS) were purchased from Sigma chemicals (USA).

### Cloning and expression of *rv0774c* in *E. coli*-*Mycobacterium* shuttle vector

The *Rv0774c* gene was amplified by PCR using forward primer AA**GGATCC**ATGATGCCCGCATGCCAGA containing BamH1 restriction site (underlined) and reverse primer AA**AAGCTT**ATAACCTGTGAGCAGCGGCGC containing HindIII restriction site to facilitate the directional cloning of gene in low copy expression vector, pVV16 containing constitutive promoter, GroEL (Kumar and Kaur, 2014). Both, pVV16 and amplified *Rv0774c* product were digested with BamH1 and Hind III restriction enzymes, followed by ligation using T4-DNA ligase (Thermo scientific, India). Ligated product was used to transform *E. coli*-DH5α cells. The recombinant plasmids were confirmed by sequencing and assigned as P_rv0774c_. The recombinant plasmids were isolated from *E. coli* clones and electroporated in electro-competent *M. smegmatis* mc^2^155 cells at 25 μF and 1.7 kV for 5 ms in Gene pulser (BioRad, USA), followed by quick addition of culture media. The culture was grown for 6 h at 37°C, followed by spreading on 7H10 agar plates (Kan+/Hyg+). Subsequently, the plate was incubated for 5 days. The expression of gene was very low to be identified on SDS-PAGE.

#### Expression and sub-cellular localization

For sub-cellular localization studies, GFP gene was cloned downstream the *rv0774c* gene sequence expressing under the GroEL promoter. The GFP sequence was amplified from the pYUBGFP plasmid using specific primers containing Hind III restriction site underlined (FWD-gfp-5′- AAGCTT ATG GTG AGC AAG GGC GAG GA-3′; REV-gfp-5′- AAGCTT ATA CTT GTA CAG CTC GTC CAT GCC G-3′). The amplified GFP was cut with Hind III restriction enzyme and ligated into P_rv0774c_ to create P_rv0774c::gfp_. The orientation of gene was confirmed by using sets of primers for GroEL promoter and GFP reverse primer followed by sequencing. Expression of green fluorescent protein (GFP) cloned downstream of the gene used to determine its expression and localization in *M. smegmatis* system. The GFP was also cloned into vector alone to create P_gfp_ and used as control. P_rv0774c::gfp_ and P_gfp_ were electroporated in *M. smegmatis* mc^2^155 cells and assigned as *Ms_rv0774c* and *Ms_ve* clones, respectively. The expression of gene was monitored by observing the expression of GFP under the fluorescent microscope. The fluorescence in extracellular media was checked by spectrofluorimetry to establish the extracellular localization of the protein. The extracellular proteins present in culture media were precipitated with 80% ammonium sulfate and dissolved in PBS. Equal amount of proteins recovered from culture media of *Ms_ve* and *Ms_v0774c* were used for spectrofluorimetry analysis (JASCO, Japan) with the excitation and emission wavelength of 480 nm and 500–530 nm, respectively, to determine the expression of GFP. Spectrofluorimeter was calibrated with culture filtrate of *M. smegmatis* mc^2^155 in order to remove background fluorescence.

#### Colony size, morphology, and growth kinetics

After 5 days of plating, the diameter of colonies was measured by GEL-DOC apparatus system (BioRad, USA). To rule out the discrepancy in morphology of bacteria based on its growth, both were grown for different time period to get nearly same size of colony. The morphology of colonies was identified by visual inspection after plating of 7 days for *Ms_rv0774c* and 10 days for *Ms_ve*. For growth kinetics, *Ms_rv0774c* and *Ms_ve* cells were allowed to grow at 180 rpm at 37°C for 27 h. The growth of cells was monitored by observing optical density at 600 nm for every 3 h interval using a spectrophotometer (GE health care, USA). Growth kinetics of *Ms_rv0774c* and *Ms_ve*, OD_600 nm_ was plotted against time and compared.

#### Drug susceptibility assay using resazurin test

The effect of Rv0774c protein on drug susceptibility was checked by using resazurin colorimetric redox indicator test (Palomino et al., 2002). The susceptibility of *Ms_rv0774c* and *Ms_ve* against the widely used anti-TB drugs, that is, isoniazid, rifampicin, and streptomycin was determined. Bacteria were grown to mid-log phase followed by diluting the cells and dispensing equal number of cells (4 × 10^5^) in 48 well plates. Drugs were diluted to working concentration ranges in 7H9 medium devoid of detergent. Drugs were added in different concentrations and incubated for 2 h. A working 1:1 dilution of 10X stock resazurin in 20% tween-80 was prepared just before use and 8.0 μL per well (48-well plate) was added. The change of resazurin into resurfin was determined by monitoring the change in color of medium from blue to pink. In a separate experiment, *Ms_rv0774c* and *Ms_ve* cells were treated with drugs of different concentrations and incubated for 2 h followed by appropriate dilution and spreading on 7H10 plate. CFUs of *Ms_rv0774c* and *Ms_ve* were counted and compared.

#### Extraction and characterization of lipids using TLC analysis

*Ms_rv0774c* and *Ms_ve* were grown to mid log phage in 250 ml 7H9 broth supplemented with 10% OADC. Cells were harvested by centrifugation at 4,000 rpm for 15 min followed by washing with PBS buffer to remove the media contamination. For lipid extraction, equal amount of pellets were dissolved in 50 ml of chloroform- methanol at a ratio of 2:1 (vol/vol) followed by 14 h constant stirring (Singh et al., 2014). The organic phage containing the lipids were separated and evaporated to dryness using rotary evaporator (IKA^@^RV10 digital, Genaxy). Lipids were dissolved in minimal amount of chloroform and characterized by TLC analysis. Polar and apolar lipids were fractionated and extracted using method of Singh et al. (2016). Briefly, glycolipids were extracted from *Ms_rv0774c* or *Ms_ve* using Methanol: Ammonium hydroxide (80:20, vol/vol) as mobile phase and detected by α-naphthol/sulfuric acid staining. The mycolic acid containing glycolipids were separated from the pool of total polar lipids using Chloroform: Methanol: Ammonium hydroxide (80:20:2 vol/vol/vol) mobile phase and visualized by Iodine vaporization.

#### Infection of THP-1 macrophages cell line

THP-1 cells (2.5 × 10^4^) were incubated in RPMI-1640 medium containing 5 ng/mL PMA in 96 well culture plates for 24 and 48 h in a CO_2_ incubator for maturation into macrophages (Takashiba et al., 1999). Cells were maintained at 37°C in 5% CO_2_ in RPMI media (Sigma Aldrich, USA) supplemented with 10% FBS and media was changed thrice, till the appendages of differentiated macrophages were seen. The differentiated cells were infected with *M. smegmatis* mc^2^155 harboring vector with *rv0774c gene*, vector alone and *M. smegmatis* mc^2^155 parental strain with different multiplicity of infection (MOI) of 10:1, 20:1, 30:1, 40:1 and 50:1. After 24 and 48 h of infection, MTT (3-(4,5-dimethylthiazol-2-yl)-2,5-diphenyltetrazolium bromide) working solution was added into the wells being assayed (20 μL for each well). Incubation was carried out at 37°C for 2 h. At the end of the incubation period, the medium was removed and formazan crystals were dissolved in 200 μL of DMSO per well. The plate was incubated for 15 min at 37°C, and absorbance was measured at 570 nm. The average of the three readings for infected cells was taken along with the control and the graph was plotted to calculate the viability.

#### Intracellular survival

Infection of *M. smegmatis* mc^2^155 carrying vector with *Rv0774c* gene *(Ms_rv0774c)* and vector alone (*Ms_ve*) in the macrophages was performed at an MOI values 1:1, 5:1, 10:1, and 20:1. The infections were allowed to proceed for 4 h at 37°C in 5% CO_2_. Extracellular bacteria were killed by incubating the cell line culture with 200 μg/mL of amikacin for 2 h. Cells were again washed twice with PBS and subsequently incubated at 37°C in fresh medium with 20 μg/mL amikacin and 50 μg/mL hygromycin for different time intervals 6, 12, 24, 48, and 72 h. At each time interval, the infected cells were washed twice with PBS, and the infected macrophage monolayers (three wells per strain) were lysed with 0.1 mL of 0.01% triton X-100 (Sigma Aldrich, USA) to release the intracellular mycobacteria. The numbers of intracellular mycobacteria were enumerated by spreading suitable dilutions onto the Middlebrook 7H10 agar plates containing Kan+/Hyg+ antibiotics.

### Immune response

#### Measurement of NO after infection

In order to determine the effect of Rv0774c on nitric oxide (NO) production in THP-1 cells, 1 × 10^5^, PMA activated THP-1 cells were infected with *Ms_rv0774c* and *Ms_ve* for 24 h. The concentration of stable nitrite, an end product from nitric oxide generation by effecter macrophages was determined by the method based on Griess reaction (Ding et al., 1988). Briefly, 100 μL of culture supernatants were incubated with an equal volume of Griess reagent (one part of 1% sulphanilamide in 2.5% phosphoric acid (H_3_PO_4_) plus one part of 0.1% naphthyl-ethylene-diaminedihydrochloride (Sigma Aldrich, USA) in water at room temperature for 10 min in a 24-well plate. The absorbance at 540 nm was then determined on ELISA plate reader (BioRad, USA). Nitrite content (μmol/mL) was quantified by extrapolation from a sodium nitrite standard curve (data not shown).

#### Estimation of cytokine levels

Culture supernatants were harvested after infection of macrophages with *Ms_rv0774c* or *Ms_ve* for 24 h. The concentrations of cytokines in the culture supernatants were determined using commercially available ELISA kits (Krishgen Biosystems, India) for tumor necrosis factor alpha (TNF-α), interferon gamma (IFN-γ), interleukin 10 (IL-10), interleukin 12 (IL-12) and MCP-1 according to manufacturer protocol.

#### qPCR analysis

Expression of *rv0774c* in recombinant *M. smegmatis* was monitored during lag phase and at 6, 12, 24, 48, and 72 h during survival assay. Total RNA was isolated using TRIzol reagent (Sigma Aldrich, USA) as per the manufacturer's protocol and cDNA synthesis was performed with 1 μg of RNAs using Revert aid cDNA synthesis kit (Fermentas, India). cDNA was used as template for real time PCR to determine transcripts of *rv0774c* with *sigA* gene as internal control. The homolog of Rv0774c was also present in *M. smegmatis* but its N- terminal region was quite dissimilar. Therefore, primers for *Rv0774c* gene (Fwd-5′-TCC TTG GCG CTA CCT CAG CAT AT-3′; Rev-5′-CCG TCA CGT AGG TGG GTG-3′) for performing real time PCR were designed from the non-identical region to ensure that the transcript measured was critically from recombinant *Rv0774c* and not from the parental genomic DNA. We also accessed the expression of nitric oxide synthase (*iNOS*) and toll like receptor 2 (*TLR2*) gene in macrophages infected with *Ms_rv0774c* or *Ms_ve* by quantitative PCR analysis. Total RNA was isolated from macrophages after bacterial infection by Trizol method (as per manufacturer's instructions) and cDNA was prepared. mRNA level of *iNOS* and *TLR2* genes were quantified using qRT-PCR (Applied Biosystems® StepOne™ Real-Time PCR Systems) using *TLR2* primers (Fwd 5′-GCCAAAGTCTTGATTGATTGG-3′; Rev5′-TTGAAGTTCTCCAGCTCCTG-3′) and *iNOS* primers (Fwd-5′-CGGTGCTGTATTTCCTTACGAGGCGAAGAAGG-3′; Rev-5′-GCTGCTTGTTAGGAGGTC AAG TAA AGGGC-3′), respectively. In each set of reactions, β-actin was used as an internal control and quantified using β-actin primers (Fwd-5′-ACC AAC TGG GAC GAC ATG GAG AAA-3′; Rev-5′-TAG CAC AGC CTG GAT AGC AAC GTA-3′).

### Estimation of TLR2 receptor protein and phosphorylation level of p38 map kinase by western blot analysis

After infection, macrophages cells were lysed in lysis buffer (10 mM Tris/HCl, 1% NP40, 50 mM NaCl, 0.1% SDS, 50 mM NaF, 50 mM DTT, 1 mM EDTA, 1 mM PMSF). Cell lysates were harvested, and total protein concentration was estimated. Equal amount of proteins of untreated macrophages (control) and treated samples were subjected to SDS-PAGE analysis. After SDS-PAGE, the proteins were transferred to the nitrocellulose membrane followed by washing and hybridization with 1:200 dilution of anti mouse TLR2 antibody, 1:200 dilution of p-p38 (D-8) mouse monoclonal IgM antibody and p38α/β (A-12) mouse monoclonal IgG1 antibody (Santa Cruz Biotechnology, USA), followed by washing and incubation with secondary conjugate anti-mouse IgG-HRP (Sigma-Aldrich, USA). Internal control β-actin was detected with anti-mouse β-actin IgG1 antibody (Santa Cruz Biotechnology, USA). After washing, blots were visualized by chemiluminescence dye Pierce™ ECL Substrate (Thermo scientific, India) and images were captured with a luminescent image analyzer. Quantification of western blot was performed by using ImageJ software (https://imagej.nih.gov/ij/), and results were expressed in terms of relative density.

#### ELISA-based analysis of serum samples of TB patients

SVMTriP tool was used to predict the antigenic epitopes of Rv0774c protein. SVMTriP achieves a sensitivity of 80.1% and a precision of 55.2% with a five-fold cross-validation. The output provides the number of eiptopes and the antigenicity score of the protein. For the pilot study, serum samples of TB patients were procured from Government Medical College and Hospital, Sec-32, Chandigarh, India. Fifteen pulmonary TB (P-TB) and 15 extra pulmonary TB (EP-TB) patients (PPD+/HIV−) were included for the present studies along with 15 healthy controls. Recombinant Rv0774c and LipY protein were expressed and purified according to protocol mentioned in our earlier work (Kumar et al., 2016). The integrity of purified protein was checked on SDS-PAGE (data not shown). Hundred micro liter of 20 μg/mL of Rv0774c and LipY (positive control) recombinant proteins were added in 96-well micro titer plates and incubated for overnight at 4°C. Fifty microgram of BSA was added in a separate well to be taken as a negative control. The remaining binding sites were blocked by incubating with 5% skimmed milk for 2 h and the unbounded constituents were removed by three washings with PBST (PBS with 0.05% tween 20). After washing, primary antibodies (1:2000 dilution in PBS of the serum samples of TB patients) were added and incubated for 2 h at 4°C followed by washing thrice with PBST. HRP- conjugated anti-human IgG secondary antibody (Sigma Aldrich, USA) was further added and incubated for 2 h followed by washing thrice with PBST and twice with PBS. Substrate OPD (o-phenylenediamine dihydrochloride) was added, and the end product was measured at 492 nm by ELISA plate reader.

### Statistical analysis

Statistical analysis of obtained results was performed by GraphPad PRISM software. We specified the mean of results performed in three independent biological replicates and ± standard deviation (SD) was also shown. Statistical significance (*P*-value) was accessed using student's *t*-test. *P*-values less than 0.05 were supposed to be statistically significant. ^*^p ≤ 0.05, ^**^p ≤ 0.01, ^***^p ≤ 0.001 was considered as statistically significant.

## Results

### Expression and sub-cellular localization of *Rv0774c* into *E. coli-Mycobacterium* shuttle vector pVV16 expression vector

**Figure 1:**
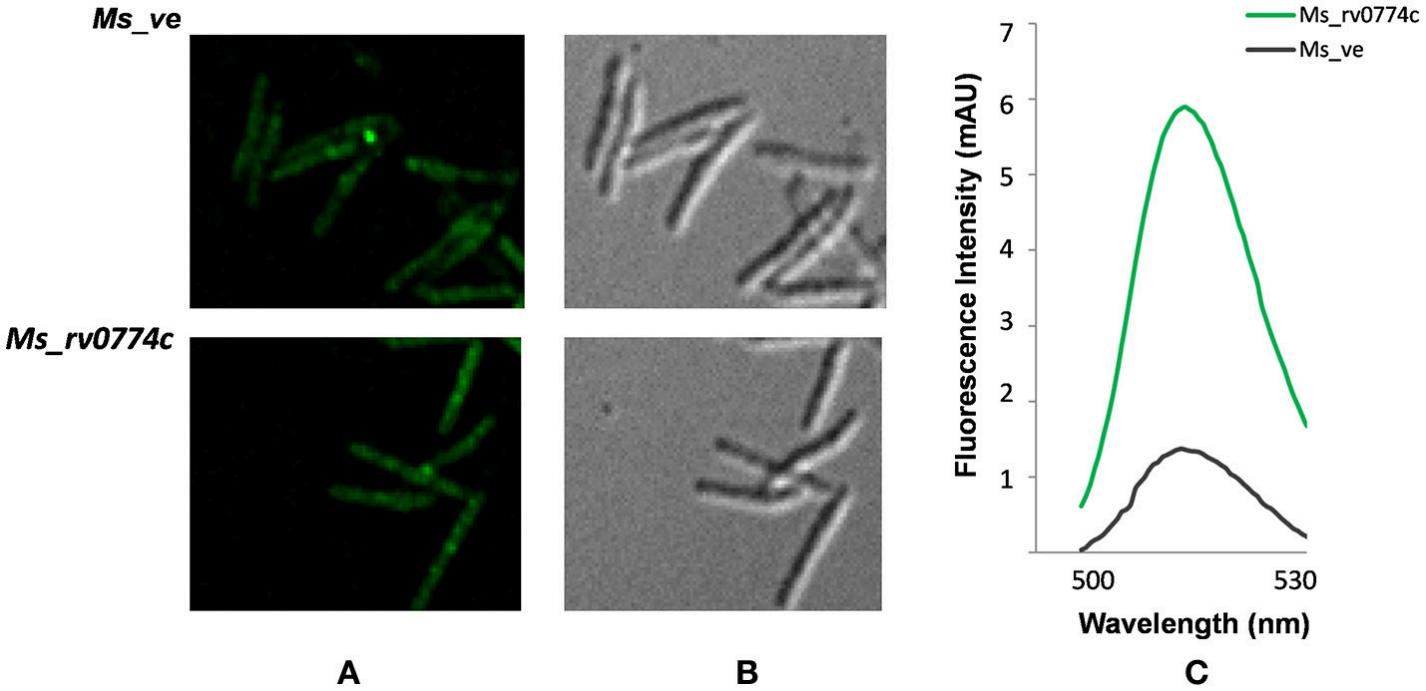
Green fluorescent protein expressed at 3′-end of *rv0774c* gene: **(A)** Image demonstrating the *M. smegmatis* expressing Rv0774c and GFP at c-terminal showing green fluorescence **(B)** Image taken without green fluorescence filter. **(C)** Expression of GFP in culture filtrate was measured by spectrofluorimetry. Green line represent fluorescence emission of culture filtrate protein of Rv0774c-GFP clone and gray line represent the GFP clone in vector alone.

Rv0774v was reported to be overexpressed/induced under several stressful conditions, encountered by the invading *M. tuberculosis* inside the hostile cellular environment (McGillivray et al., 2015; Kumar et al., 2017). Therefore, we chose pVV16 a multicopy constitutive vector than the single copy vector for this study. The *Rv0774c* gene was expressed in *M. smegmatis* mc^2^155 cells. Host with vector alone was used as a control. In order to identify the expression of Rv0774c protein, GFP protein was fused at the c- terminus of Rv0774c (*Ms_rv0774c*) and was present in vector alone *(Ms_ve)*. As the GFP was present at the c- terminal region of the gene *Rv0774c*, the expression of GFP was directly proportional to the Rv0774c expression. The expression of the gene was confirmed by fluorescence microscopy. The transformed rod shaped cells were showing green fluorescence (Figures 1A,B). The GFP was present in culture filtrate protein of *Ms_rv0774c*, whereas, no fluorescence was observed in *Ms_ve* (Figure 1C) confirming that Rv0774c is secreted out into the media.

### Colony size, morphology, growth curve analysis of *Ms_rv0774c* and *Ms_ve*

**Figure 2:**
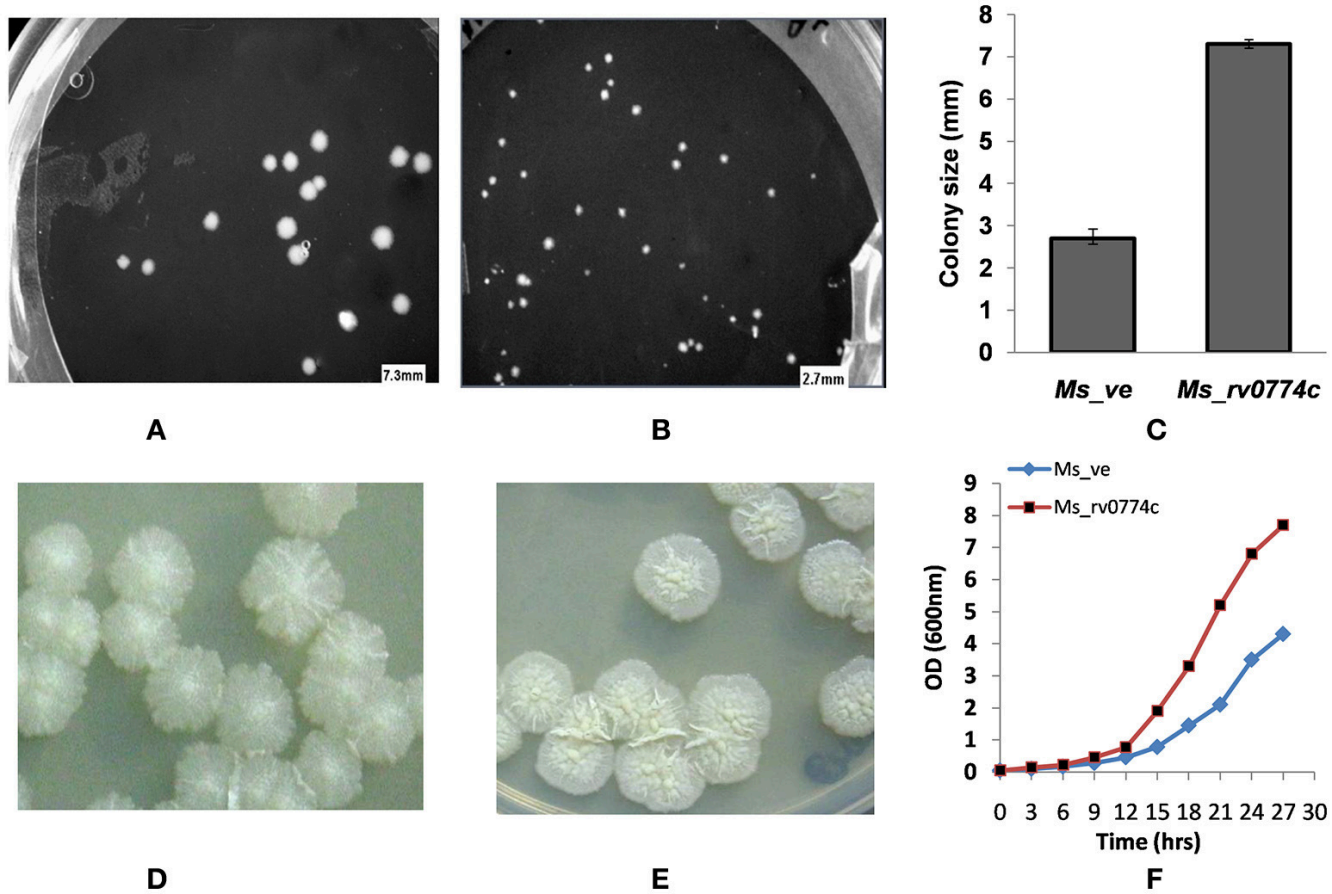
Effect of expression of *rv0774c* on colony size, morphology, and its growth: colony size of the *M. smegmatis* expressing *rv0774c* (*Ms_rv0774c*) **(A)** and containing vector alone (*Ms_ve*) **(B)**. **(C)** Average diameter (mm) reflecting the size of *Ms_rv0774c* and *Ms_ve* on day 5 of incubation at 37°C. Colonies morphology **(D)** *Ms_rv0774c* **(E)** *Ms_ve*. Colony images were digitally captured by Phase Contrast Microscope (Olympus, USA). **(F)** Effect of *rv0774c* expression on growth curve of *M. smegmatis*: *Ms_rv0774c* has shown rapid growth in lag phase as compared to *Ms_ve*. Each dot represents mean OD of three individual experiments.

The colonies of the *Ms_rv0774c* appeared on the solid agar based medium after 3 days of plating, while the colonies transformed with vector alone (*Ms_ve*) appeared after 4 days (Figures 2A,B). Colonies of *M. smegmatis* mc^2^155 transformed with *Rv0774c* appeared to be about 3.0 fold larger than control (Figure 2C). The colony morphology of *Ms_rv0774c* was also quite distinct than control. The recombinant colony of *Ms_rv0774c* showed unusual colony shape. The colony surface of recombinants was smooth, wet and flat in comparison to control that was spherical, frill and with obvious bulging (Figures 2D,E). *Ms_rv0774c* showed a short lag phase in comparison to *Ms_ve*. *Ms_ve* exhibited a uniform growth rate till the cell culture reached the stationary phase of growth (Figure 2F).

### Susceptibility of *Ms_rv0774c* against the anti TB drugs

**Figure 3:**
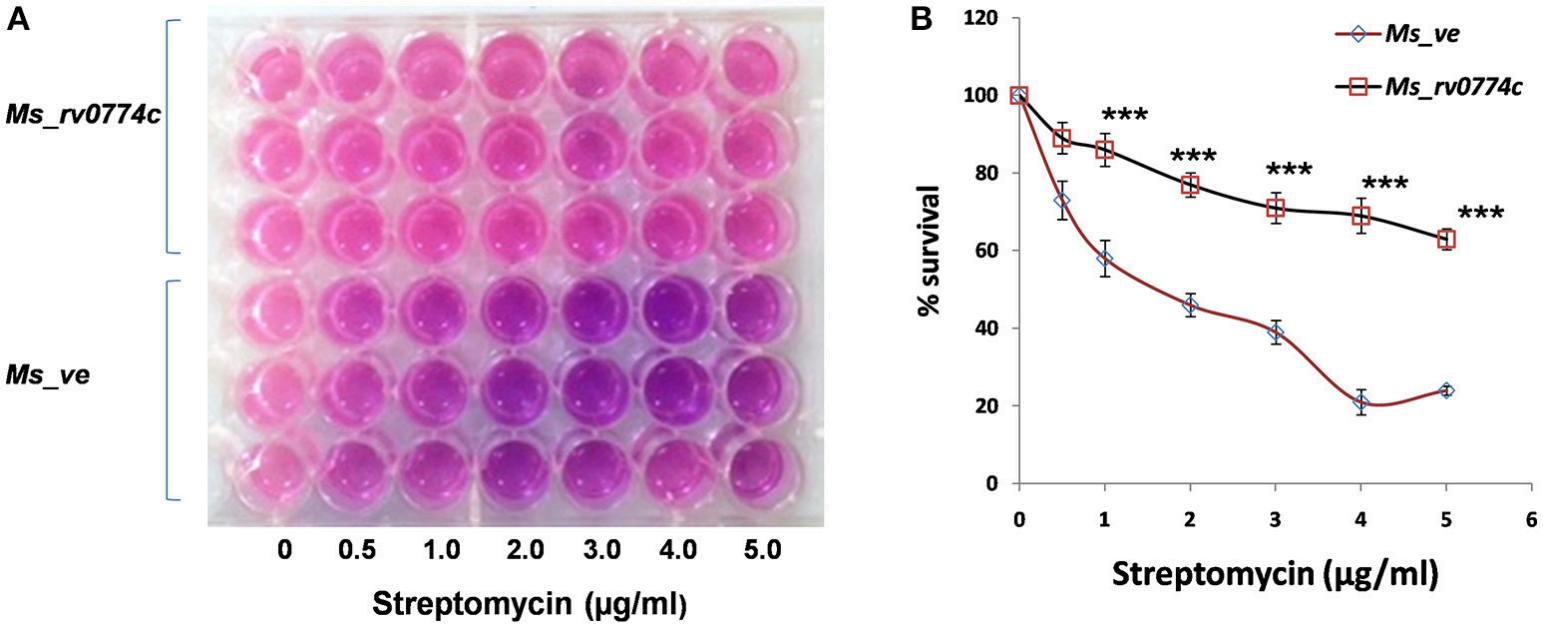
Effect of Rv0774c on drug susceptibility of *M. smegmatis*: **(A)** Mid log-phase *M. smegmatis* culture diluted in 7H9 medium without tween 80 and were treated with indicated concentrations of streptomycin. Resazurin dye in 20% tween 80 was added after 3 h of incubation with streptomycin, and the color change was monitored after 24 h. Pink color indicates live bacteria, while blue indicates dead bacteria **(B)** Survival of *Ms_rv0774c* and *Ms_ve* was monitored by counting the CFU/mL after treatment of streptomycin. Results were expressed in % survival (CFU counts without drug treatment was considered to be the 100% survival). Data are representative of three independent biological replicates and shown as mean ± *SD*. Statistical analysis was assessed using student's *t*-test (^***^P ≤ 0.001).

The effect of Rv0774c on drug susceptibility of *M. smegmatis* was monitored in the presence of well-known anti-TB drugs such as isoniazid, rifampicin, and streptomycin. Treatment of *Ms_rv0774c* with streptomycin resulted in significant enhancement in survival of *Ms_rv0774c* as compared to *Ms_ve. Ms_rv0774c* showed resistance up to 5 μg/mL of streptomycin (Figures 3A,B; Supplementary Table 1). However, no significant change was observed in case of other anti-TB drugs (Data not shown).

#### *Ms_rv0774c* modulate the trehalose di mycolate (TDM) content of cell wall

**Figure 4:**
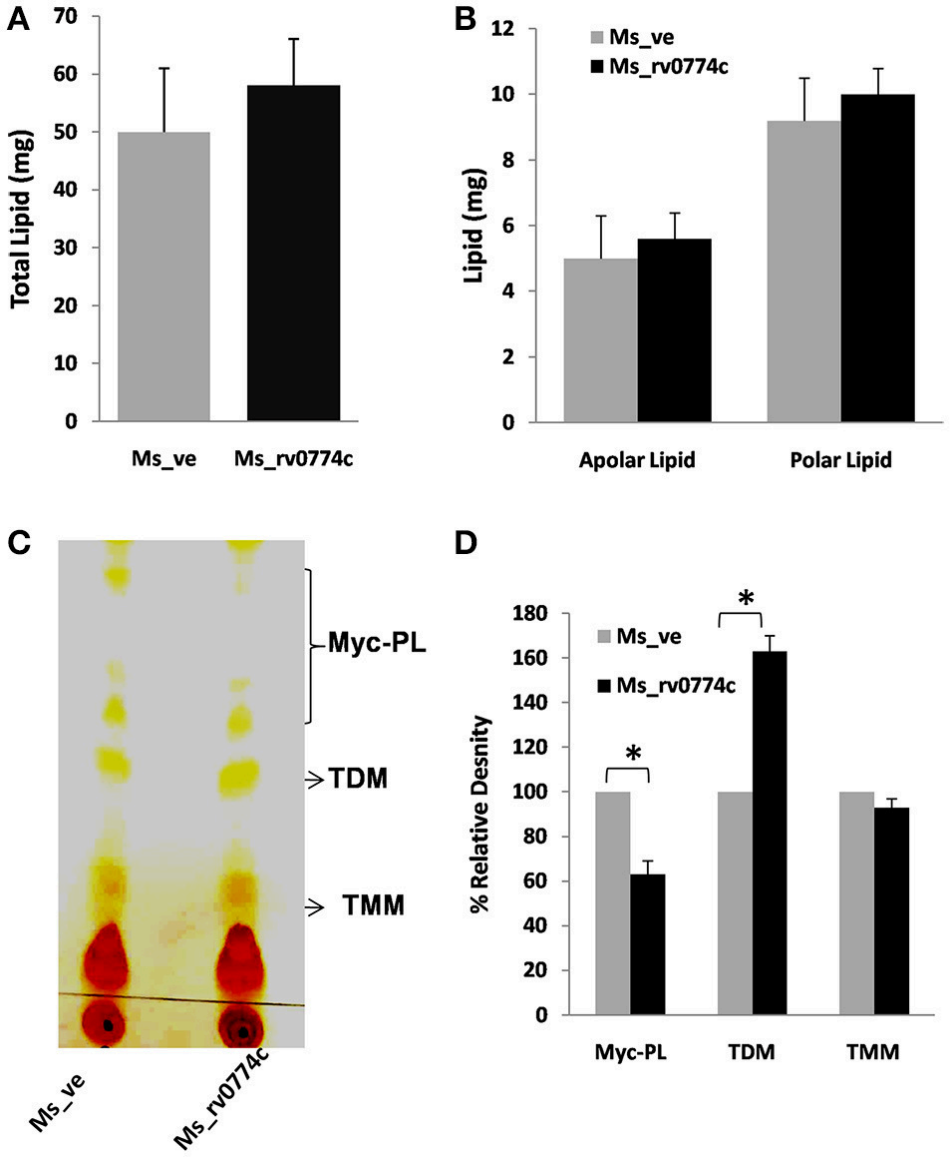
Modulation of mycolipids in *Ms_rv0774c*: Quantitative estimation of **(A)** total lipids, **(B)** polar and apolar lipids extracted from the equal weight of *Ms_ve* or *Ms_rv0774c*. **(C)** Mycolic acid containing glycolipids were separated from the pool of total polar lipids using CHCl_3_:CH_3_OH:NH_4_OH (80:20:2) as mobile phase. **(D)** TLC results were expressed in term of % relative density of myco-glycolipids measured by Image J programme. Given data are expressed in mean ± *SD* performed in two independent experiments. Statistical analysis was assessed using student's *t*-test (^*^P ≤ 0.05).

The changes in colony morphology and increased drug susceptibility were reported to be associated with the remodeling of components of the cell wall lipids (Deng et al., 2015; Singh et al., 2016). Therefore, we analyzed the lipid content of Ms_ve and *Ms_rv0774c* cells at the same stage of growth. *Ms_rv0774c* was found to have slight increased quantities of total lipids in the cell wall. However, the polar and apolar lipid content was not found to be significantly changed (Figures 4A,B). Compare to Ms_ve, total glycolipids were also not changed in *Ms_rv0774c* (data not shown). We also analyzed the effect of Rv0774c on cell wall mycolate containing glycolipids [trehalose monomycolate (TMM), trehalose dimycolate (TDM) and mycolylmannosylphosphorylheptaprenol, (Myc-PL)]. TLC results indicated an increase of TDM and decrease of Myc-PL in *Ms_rv0774c* cell wall. However, the relative amounts of TMM remain unchanged (Figures 4C,D). TLC analysis of mycolate containing glycolipid corroborated that *Rv0774c* might not alter the total lipid content but was involved in remodeling of lipids.

### Determination of multiplicity of infection (MOI) and intracellular survival of *Ms_rv0774c* and *Ms_ve* in human macrophages cell lines

**Figure 5:**
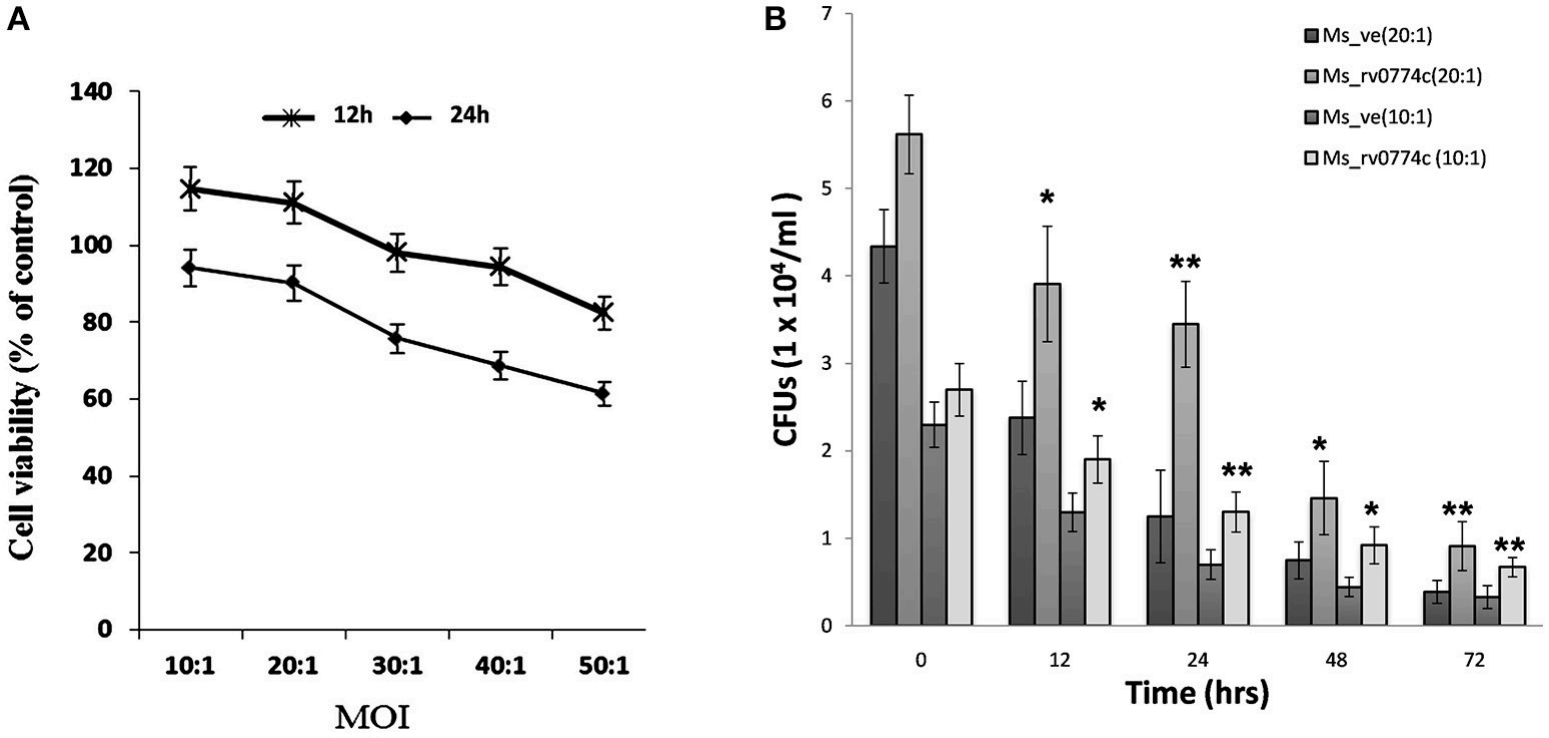
**(A)** Viability of THP-1 macrophages cell after infection. Viability assay showed that *Ms_rv0774c* has little effect at MOI of 10:1 and 20:1 for 24 h of infection, while significantly decreased viability was observed at higher MOI of 30:1, 40:1, 50:1, and 48 h of infection **(B)** Intracellular survival of *Ms_ve* and *Ms_rv0774c*. CFU counts at different time intervals after infection of THP-1 macrophages with *Ms_ve* or *Ms_rv0774c* at MOI values 10 and 20. Data are representative of three independent biological replicates and shown as mean ± *SD*. Statistical analysis was assessed using student's *t*-test (^*^P ≤ 0.05 and ^**^P ≤ 0.01).

To determine the multiplicity of infection of THP-1 macrophages with recombinant clone, THP-1 macrophages were infected by recombinant *Ms_rv0774c* or the vector alone strains at different MOI. For 24 h of infection at MOI of 10:1, 20:1, 30:1, 40:1, and 50:1, the viabilities of macrophages were 120, 113, 101, 97, and 86%, respectively, for *Ms_rv0774c*, compared to the *Ms_ve*. It showed macrophages cells are more viable at lower MOI value up to 20:1, however, further rise of MOI led to decline in viability, indicating that the recombinant *M. smegmatis, Ms_rv0774c* can inhibit the viability of THP-1 macrophages at higher MOI values. After 48 h of infection, the macrophage viability was 89.1, 85.6, 78.1, 69.3, and 59.4%, respectively, compared to the control strain (Figure 5A). Results suggested that the THP-1 cells were more viable at lower MOI value after 24 h of infection.

*M. smegmatis* is inherently unable to multiply inside macrophages and the number of intracellular bacteria decreased gradually after infection of macrophages *in ex vivo*. In order to determine whether Rv0774c facilitated the intracellular survival of these bacteria in macrophages, we compared the survival kinetics of *Ms_rv0774c* and *Ms_ve* in THP-1 macrophages at different MOI values. The results showed significant 2-folds difference in the number of intracellular bacteria between *Ms_rv0774c* and *Ms_ve* after 24 h infection, while nearly 1.6–1.8 folds enhanced survival was noticed in *Ms_rv0774c* in comparison to *Ms_ve* after 72 h of infection with MOI values 10 and 20. However, at low MOI values 1:1 and 1:5, the changes were not statistically significant (data not shown). These results suggested that the presence of Rv0774c was able to enhance the intracellular survival of *M. smegmatis* in macrophages (Figure 5B).

### *M. smegmatis* expressing *rv0774c* reduced the production of reactive nitrogen species (NO), *iNOS* expression and induced TLR2 expression in macrophages cell line

**Figure 6:**
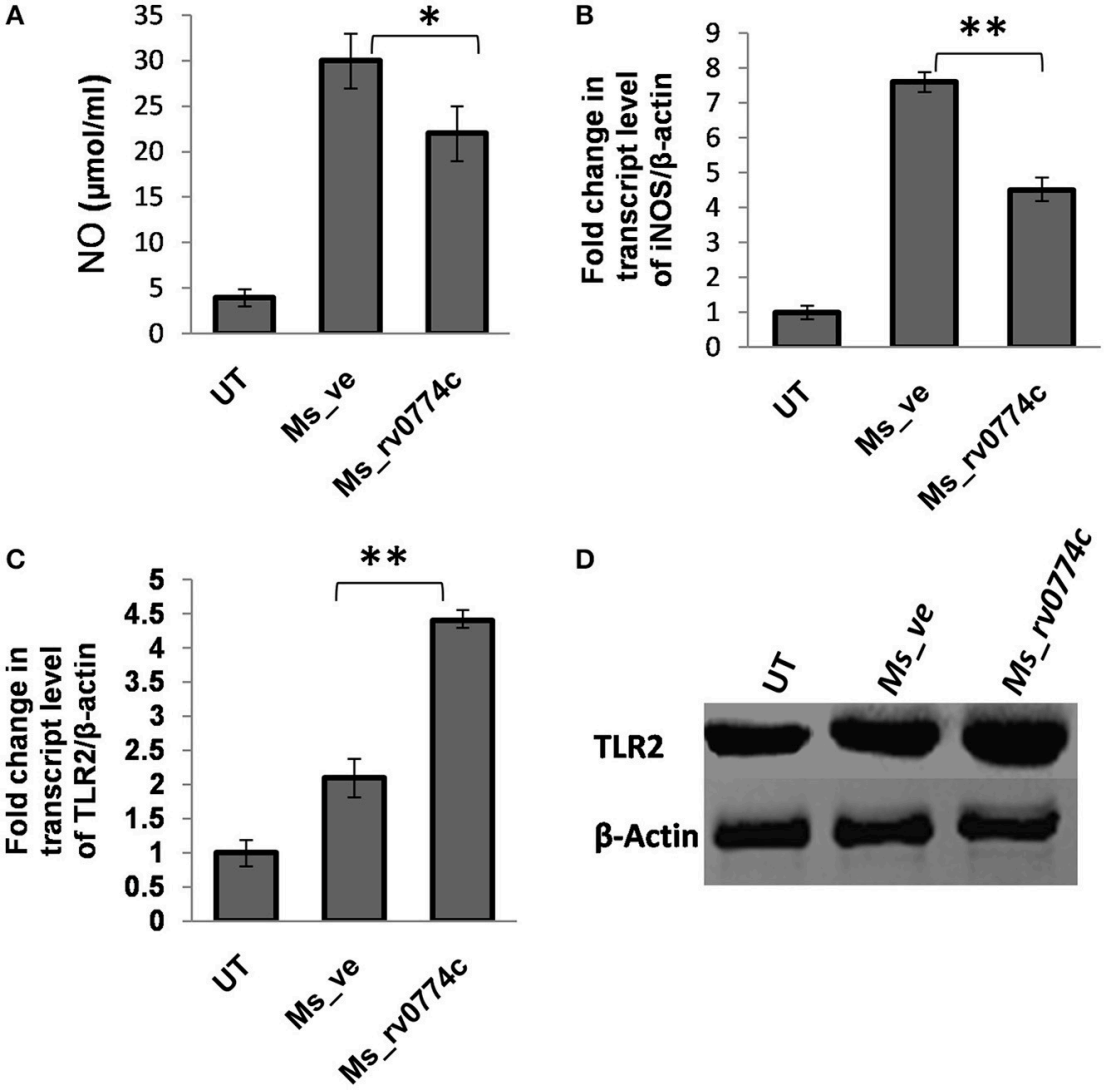
**(A)** Measurement of NO secretion in THP-1 cells after infections of macrophages with Ms_ve or Ms_rv0774c for 24 h. **(B)** Transcript level of *iNOS* after infections was determined by qPCR. **(C)** The expression of *TLR2* transcript was examined by real time PCR using specific primers after infection. **(D)** Expression of TLR2 was determined by western blot. Total proteins were isolated from THP-1 cells after infection. After electrophoresis the proteins were transferred onto nitrocellulose membrane and developed with antibodies against TLR2 and β-actin (internal control). Data are representative of three independent biological replicates and shown as mean ± *SD*. Statistical analysis was assessed using student's *t*-test (^*^P ≤ 0.05 and ^**^P ≤ 0.01).

Significant survival of *M. smegmatis* mc^2^155 expressing *rv0774c* in comparison to control suggested that Rv0774c might be involved in immune modulation of infected macrophages. The results were significant at MOI values 10 and 20, but this was more prominent at MOI 20. Rv0774c is a stress induced protein under its native promoter and in pVV16 it is expressed under weak constitutive promoter. In addition it is a secretory protein. Therefore, a particular level of protein might be required for immune modulation and thus increasing the number of bacteria during infection (MOI 20) might have increased the probability of Rv0774c to modulate the immune response significantly. Therefore, for immunological studies, we used MOI value 20. We examined the effect of Rv0774c on production of reactive nitrogen species (NO), a primary cytotoxic weapon utilized by immune defense to kill intracellular mycobacteria. Result showed that the NO production in *Ms_rv0774c* infected macrophages was around 30% lower than *Ms_ve* infected macrophages (Figure 6A). Production of NO is directly correlated with the expression of *iNOS*. The expression of *iNOS* in *Ms_rv0774c* infected macrophages was lower than the macrophages infected with *Ms_ve* suggesting that the higher intracellular survival of *Ms_rv0774c* might be associated with lowered production of NO/*iNOS* expression (Figure 6B). We accessed the expression of TLR2 protein through western blot analysis. After infection, the expression of TLR2 was induced but comparatively more expression was observed in *Ms_rv0774c* (Figures 6C,D).

### *M. smegmatis* expressing *Rv0774c* suppressed the pro-inflammatory response in macrophages cell line

**Figure 7:**
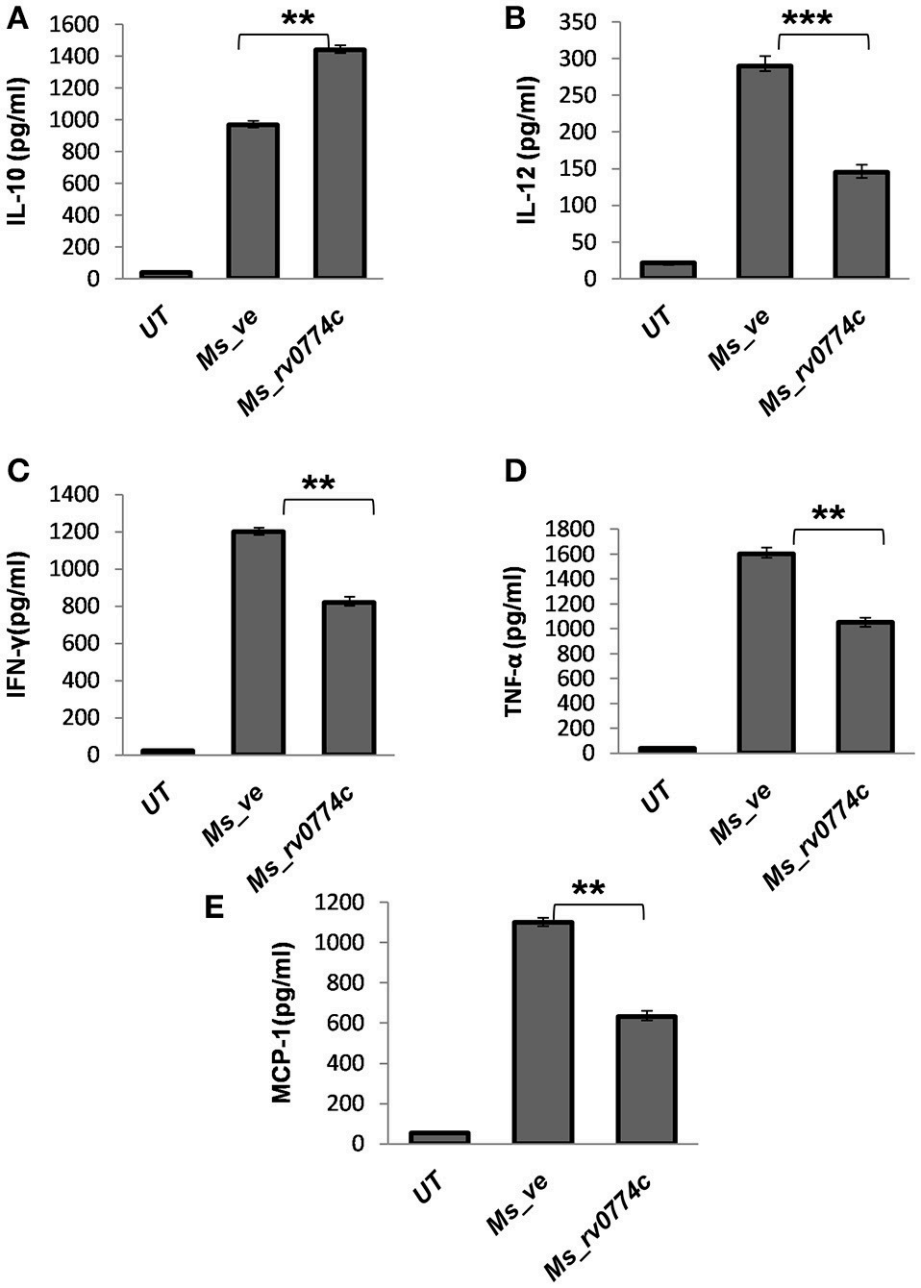
Ms_rv0774c manipulates the production of anti and pro-inflammatory cytokines by THP-1 macrophage cells: THP-1 macrophage cells were infected with Ms_rv0774c or Ms_ve at an MOI of 20. Culture supernatants were harvested after 24 h of infection and the concentrations of IL-10 **(A)**, IL-12 **(B)**, IFN-γ **(C)**, TNF-α **(D)**, and MCP-1 **(E)** were measured by ELISA. Data shown as mean ± *SD* and representative of three independent biological replicates. Statistical analysis was assessed using student's *t*-test (^**^P ≤ 0.01 and ^***^P ≤ 0.001).

Generally, TLR2 initiated signaling cascade lead to the generation of immune response. Therefore, we also estimated the production of cytokines in infected macrophages. Compared to *Ms_ve*, the *Ms_rv0774c* produced 33% more IL-10 in macrophages. However, the production of pro-inflammatory cytokines such as IL-12, TNF-α, and IFN-γ by *Ms_rv0774c* was comparatively much lower than macrophages infected with *Ms_ve*. *Ms_rv0774c* infected macrophages produced 35% lesser amount of IFN-γ and TNF-α while around 50% reduction in IL-12 production as compared to *Ms_ve* infected macrophages. Results indicated that the Rv0774c enhanced the anti-inflammatory response and down regulated the pro-inflammatory response (Figure 7). Also, *Ms_rv0774c* infected macrophages produced lower amount of MCP-1 than *Ms_ve* infected macrophages.

### *M. smegmatis* expressing *Rv0774c* lowered the level of p38 MAPK phosphorylation

**Figure 8:**
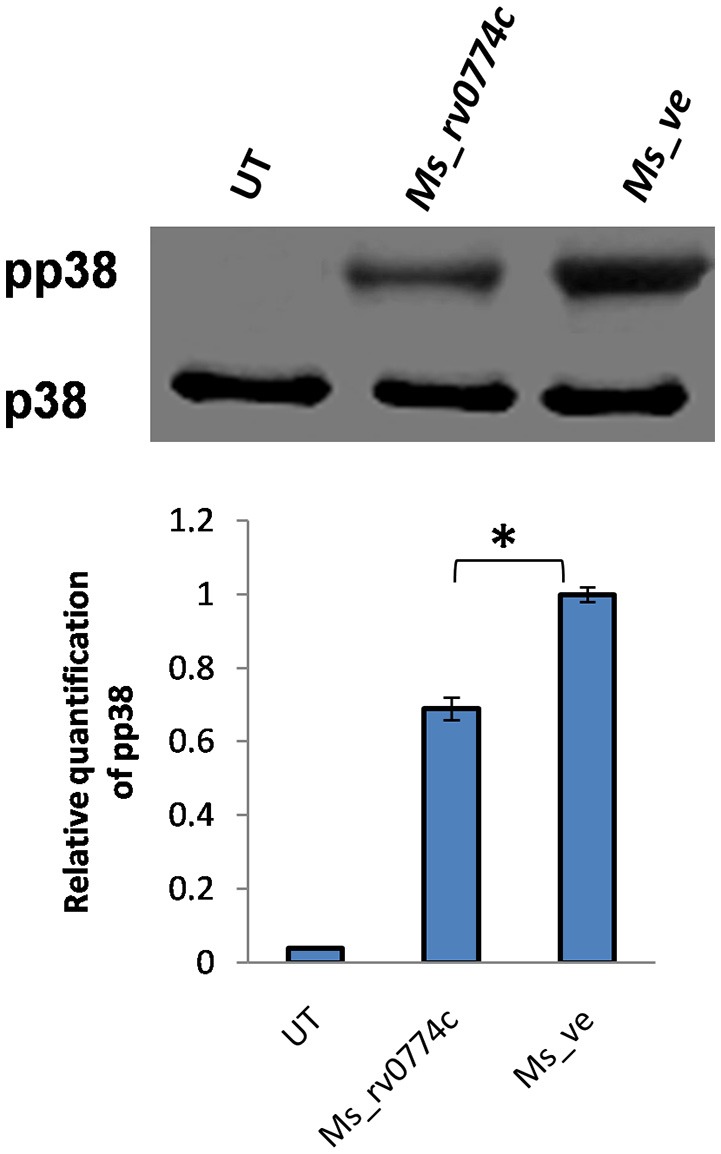
*M. smegmatis* expressing *rv0774c* has shown reduction in phosphorylation level of p38MAPK: After infection of activated THP-1 cells, the proteins were isolated, estimated, and immunoblotted by probing with the phosphorylated and unphosphorylated p38 antibody. Density of expressed proteins was quantified and expressed in relative change in phospho-p38 expression. Results shown as mean ± *SD* are the representation of three independent biological replicates. Statistical analysis was assessed using student's *t*-test (^**^P ≤ 0.05).

It was previously reported that IL-10 expression induced immune response is associated with the MAP kinase signaling cascade (Nair et al., 2009). After infection, the levels of un-phosphorylated and phosphorylated p38 MAPK were examined by western blot analysis. The levels of phosphorylated p38 in *Ms_rv0774c* were relatively lower than that observed in *Ms_ve* infected macrophages (Figure 8).

### Detection of IgG antibodies against Rv0774c in serum samples of TB patients

**Figure 9:**
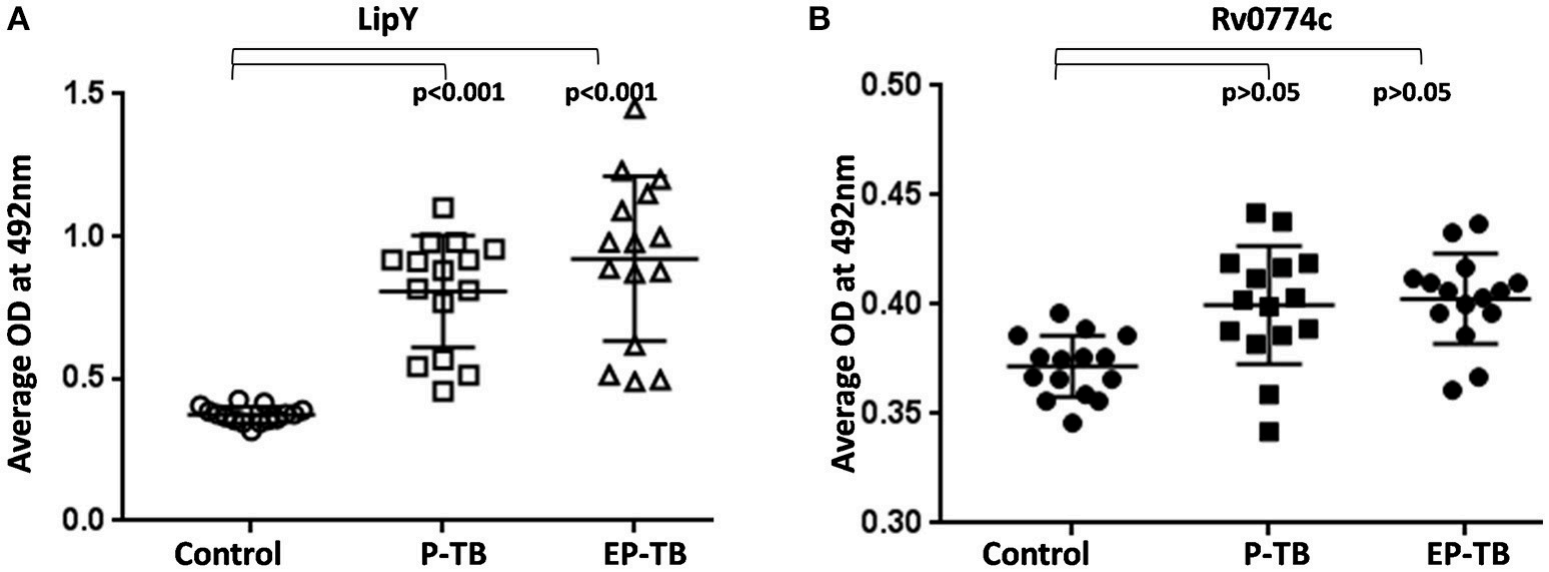
IgG reactivity for serum samples of TB: LipY **(A)** and Rv0774c **(B)**. P-TB and EP-TB represent pulmonary and extra pulmonary TB. Experiments were performed three times in triplicates. Each dot represents mean OD of three individual experiments for individual patient. Statistical analysis was assessed using student's *t*-test.

Rv0774c was identified in the culture filtrate of *M. tuberculosis*, showing its secretory nature. *In silico* analysis revealed three potential antigenic epitopes in Rv0774c with high score (Supplementary Table 2). Therefore, we checked the presence of IgG serum antibodies against the Rv0774c protein in active serum sample of TB patients. The reactivity of LipY, a PE containing lipase has shown reactivity with sera of patients with both pulmonary (P-TB) and extra-pulmonary TB (EP-TB) (Mishra et al., 2008). So, we used LipY as a positive control in our experiments, and it exhibited reactivity with the sera of both patients with P-TB and EP-TB (Figure 9A). The reactivity of Rv0774c was also measured in the serum samples of patients with TB and healthy individuals. Despite the secretory nature and presence of antigenic epitopes, Rv0774c did not show any significant reactivity with the serum samples of patients with TB when compared with control (Figure 9B).

## Discussion

*M. tuberculosis*, an intracellular pathogen, evolved various strategies to survive within the host (Gengenbacher and Kaufmann, 2012). All events are highly regulated by proteins encoded by the genome of mycobacteria. The difference in cell size and colony morphology of *M. smegmatis* recombinant strain expressing *Rv0774c* was consistent with the findings of Delogu et al. (2004) and Singh et al. (2016). The expression of the *Rv1818c* gene and *Rv1169c* resulted in a significant modification of the colony size and morphology phenotype in *M. smegmatis*. Colony size of *M. smegmatis* expressing PE domain of Rv1818c were larger than that containing vector alone and were showing smooth morphology (Delogu et al., 2004). Similarly, the expression of *Rv1169c* in *M. smegmatis* resulted in larger, round, shiny, and smooth colony, while control colonies were comparatively smaller, drier and more fragile. The change in size and morphology of *M. smegmatis* expressing *Rv1169c* was attributed to alteration in cell wall lipids due to its lipolytic nature (Singh et al., 2016). Despite this, *M. smegmatis* expressing *Rv1169c* was also more resistant to various environmental stresses and antibiotics. Our data also pointed out that Rv0774c might be involved in the cell wall lipid remodeling that conferred altered morphology, increased streptomycin resistance and intra survival advantages to the mycobacteria. Our previous studies showed that Rv0774c belongs to the lipase family (Kumar et al., 2016, 2017) pointing toward a possibility that it might affect cell wall lipids that led to the change in colony morphology of *M. smegmatis*. Therefore, we analyzed its role in lipid modulation. *M. smegmatis* expressing *Rv0774c* did not show any change in amount of glyco-lipids and polar lipids of cell wall. However, expression of *Rv0774c* caused a significant increase of TDM content along with noticeable decrease in Myc-PL. These results suggested that the Rv0774c might be involved in mycolation of TMM to form TDM at surface using Myc-PL. However, it is yet to be determined how secretary Rv0774c protein is involved in the formation of TDM from Myc-PL. Previously, Takayama et al. (2005) reported that Rv0774c has shown significant sequence similarity of the secretory Ag85 complex proteins of *M. tuberculosis*, which were reported to be involved in transfer of mycolic acid to TMM to form TDM (Takayama et al., 2005). Our TLC analysis also suggested that Rv0774c might act as mycolyl transferase II as predicted earlier by Takayama et al. (2005) and transfer mycolate molecule leading to deposition of TDM at surface. Similarly, increase in TDM in cell wall of *M. smegmatis* expressing PE11 resulted in smooth colony and increase in size (Singh et al., 2016). Tolerance of *Ms_rv0774c* against streptomycin might correlate with deposition of TDM at cell surface, however intolerance against other antibiotics is yet to be revealed. In a previous study, Ramón-García et al. (2006) constructed genomic libraries of DNA of *M. fortuitum* and found that a 2.5 kb fragment possessing 5 reading frames has 64-fold resistance to streptomycin and has sequence identity with Rv0774c protein of *M. tuberculosis* (Santiago Ramón-García et al., 2006). Elena and others in 2013 reported that mutation in Rv0774c increased the susceptibility of *M. tuberculosis* Russian strain against anti-TB drugs including streptomycin. However, they have not specified its role in streptomycin resistance (Ilina et al., 2013).

A mounting body of evidence indicated that the bacterial components facilitate the intracellular bacteria to survive in host cell by circumventing its fate on target cells (Deretic, 2008; Gengenbacher and Kaufmann, 2012). As *Ms_rv0774c* did not induce cell death at lower MOI values during early point of infection, we hypothesized that the inhibition of apoptosis by Rv0774c might favor an adaptive mechanism for intracellular survival of bacteria, while it may be beneficial for the host to initiate an effective immune response against *Mycobacterium* infection. The enhanced intracellular survival in presence of Rv0774c indicated its potential role in bacterial persistence. In innate immune response, the cells produce excess NO and induce the expression of *iNOS* in response to mycobacterium infection (van Crevel et al., 2002). Expression of *Rv0774c* was also able to decrease the NO production and *iNOS* expression in macrophages. The present study is also in line with the *M. smegmatis* expressing *Rv0285, Rv1386*, and *Rv0256c* (PPE2), which demonstrated inhibition of iNOS/NO production and survived significantly longer inside macrophages (Tiwari et al., 2012; Bhat et al., 2017). Protein-protein interaction analysis showed the co-occurrence of Rv0774c with LprG protein of *M. tuberculosis*. Similar to Rv1411c protein (Drage et al., 2010), we reported that Rv0774c contains hydrophobic pocket for binding of lipids extended through a large cavity (Kumar et al., 2016), which might have possible binding site for TLR protein also. The enhanced expression of TLR2, with no change in expression of TLR4, might have resulted in decreased production of pro-inflammatory cytokines and was similar to our earlier report (Kumar et al., 2017). Therefore, it was hypothesized that higher survival rate might be associated with the decreased host defense phenomenon. Previously reported Rv1808 (PPE32) and Rv1196 (PPE18) protein induced the expression of TLR2, which resulted in increased secretion of anti-inflammatory cytokine, IL-10 (Nair et al., 2009; Deng et al., 2014). Similar to this finding, our result also corroborated that expression of *Rv0774c* in *M. smegmatis* resulted in increased IL-10 and decreased IL-12 production. IL-10 is known for its inhibitory role in the production of cytokines involved in pro-inflammatory response. Increased IL-10 production inhibited the production of IL-12 in macrophages treated with recombinant Rv1196 protein and infected with *M. smegmatis* strains expressing *Rv0285* and *Rv1386* (Bhat et al., 2012; Tiwari et al., 2012). Monocyte Chemo-attractant Protein-1 (MCP-1), a chemokine is actively engaged in the formation of granuloma by recruiting the macrophages at the infection site (Ameixa and Friedland, 2001). Lower production of MCP-1 protein, IL-12, TNF-α, and IFN-γ and increased production of IL-10 in response to *Rv0774c* expression suggested its immune suppressive role for facilitating mycobacterium survival. Our results also corroborated that the suppression of pro-inflammatory cytokines is most likely associated with decreased phosphorylation of p38 MAPK. Similarly Chandra and Naik reported that *Leishmania donovani* stimulated the expression of IL-10 expression via MAPK pathway by suppressing pp38 (Chandra and Naik, 2008). The results of immune modulation by purified recombinant Rv0774c demonstrated variation (Kumar et al., 2017) as the two systems used were very different in terms of availability of protein, transportation and amount of protein.

It was previously reported that the secretory proteins of *M. tuberculosis* could be the potential biomarkers for the diagnosis of active and latent tuberculosis. The presence of IgG serum antibodies against the *Mycobacterium* secretory proteins was identified in the serum of the patient with TB (Zhang et al., 2015). Earlier report also revealed that proteins of PE/PPE multigene family of *M. tuberculosis* elicited the level of both humoral and cell mediated immune response (Narayana et al., 2007; Tundup et al., 2008; Chaturvedi et al., 2010). As Rv0774c was identified in extracellular milieu and good numbers of antigenic epitopes were predicted by bioinformatic analysis, attempts were made to determine the humoral response of Rv0774c in patient samples. No significant level of IgG in active TB patient serum against Rv0774c suggested that it did not alter the humoral response.

Overall the expression of *rv0774c* in *M. smegmatis* led to changes in its intracellular survival in macrophages and altered immune response. The enhanced anti-inflammatory response might be one of the factors for bacterial persistence in macrophages and therefore, it could be the good drug target for treatment of tuberculosis. However, the interaction of different pathways in *M. smegmatis* is entirely different than the *M. tuberculosis*. Therefore, the relevance of its role in virulent *M. tuberculosis* could be confirmed by gene knock out and animal studies.

## Conclusion

These findings indicated a potential role of Rv0774c in altered morphology of bacterial colonies, resistance to streptomycin, immune-modulation and enhanced intracellular survival of *M. smegmatis* in THP-1 macrophages cell line. Rv0774c might be involved in mycolation of TMM to form TDM at surface using Myc-PL. Further the probability of Rv0774c to participate in the strategies employed by mycobacterium to avoid the extremely hostile environment inside the macrophages to support its intracellular survival cannot be ruled out. However, direct evidence is required to support/refute its role in *M. tuberculosis* H37Rv.

## Author contribution

Experiments performed and wrote manuscript: ArbindK. Analysis of Immunological experiments: AnjaniK. TB patients Sample collection, classification and characterization: VS and JasbinderK. Conceived or designed the experiments and wrote manuscript: JagdeepK.

### Conflict of interest statement

The authors declare that the research was conducted in the absence of any commercial or financial relationships that could be construed as a potential conflict of interest.
