# Supplementary material for: Modulation of Trehalose Dimycolate and Immune System by Rv0774c Protein Enhanced the Intracellular Survival of Mycobacterium smegmatis in Human Macrophages Cell Line
Source: Front Cell Infect Microbiol. 2017 Jun 30;7:289. doi: 10.3389/fcimb.2017.00289 (PMC5491638; doi:10.3389/fcimb.2017.00289)
Supplement: Supplementary file 1 [file Table1.pdf]

Supplementary Table 1: CFUs counts after "Streptomycin treatment of *Ms_ve* and *Ms_rv0774c*"

		Streptomycin (2 hrs of treatment) (CFUsx10^5/ml)					
	**0.0 µg/ml**	**0.5 µg/ml**	**1 µg/ml**	**2 µg/ml**	**3 µg/ml**	**4 µg/ml**	**5 µg/ml**
**Ms_rv0774c**	5.11±0.20	4.71±0.31	4.40±0.29	3.92±0.19	3.65±0.32	3.46±0.27	3.24±0.17
**Ms_ve**	4.70±0.37	3.59±0.46	2.76±0.34	2.17±0.39	1.81±0.18	1.12±0.14	1.14±0.04
