# Supplementary material for: Modulation of Trehalose Dimycolate and Immune System by Rv0774c Protein Enhanced the Intracellular Survival of Mycobacterium smegmatis in Human Macrophages Cell Line
Source: Front Cell Infect Microbiol. 2017 Jun 30;7:289. doi: 10.3389/fcimb.2017.00289 (PMC5491638; doi:10.3389/fcimb.2017.00289)
Supplement: Supplementary file 2 [file Table2.pdf]

Supplementary Table 2: Epitopes prediction of "**Rv0774c** and **LipY** protein"

	**Epitopes sequence**	**Score**
**Rv0774c**	DTQRLDTSRVAFLG	1.000
	ALGSIPIRVDCGNS	0.767
	MARMPELSRRAVLG	0.577
	CAVSPALWLSAGSV	0.456
	GGYGALLLGSRLGP	0.415
	GSFVSAARAGKMTN	0.390
	GSASAVMDGGVEQG	0.384
	AGTVLGATSAYAID	0.380
	PFAVVSVDGGSSYW	0.366
	LPAELTWFAPLLTG	0.362
**LipY**	LLSLSALETHFAIP	1.000
	DEVSAAIAALFSGH	0.672
	SYVVALPEVMSAAA	0.527
	PSSMVLLSPWLDVG	0.523
	HGVSNVSVVGDSAG	0.513
	NASLLQSEFASGIG	0.508
	TDVASIGSVVATAS	0.501
	AVFHERFVQALTGA	0.477
	PLLNSLLGQTVQYT	0.449
	PLLALIASDIPPLS	0.436
