# Supplementary figures and images for: Modulation of Trehalose Dimycolate and Immune System by Rv0774c Protein Enhanced the Intracellular Survival of Mycobacterium smegmatis in Human Macrophages Cell Line
Source: Front Cell Infect Microbiol. 2017 Jun 30;7:289. doi: 10.3389/fcimb.2017.00289 (PMC5491638; doi:10.3389/fcimb.2017.00289)

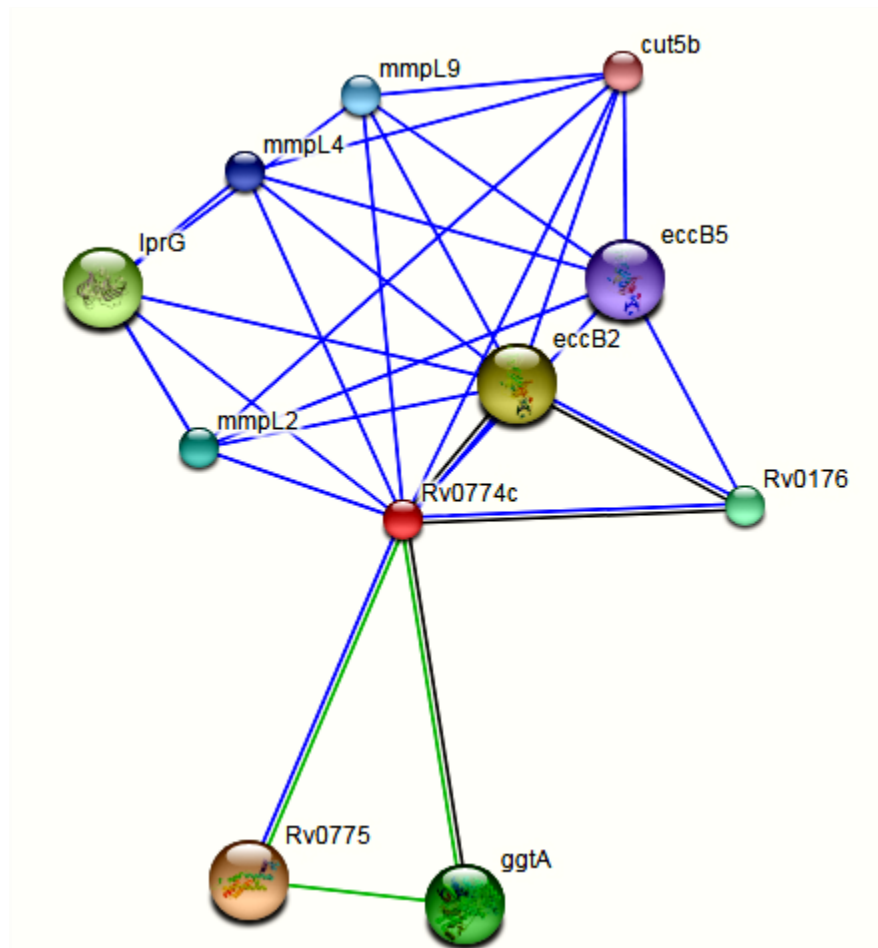

**Supplementary Figure 1:** Protein-protein interaction analysis of Rv0774c using STRING tool.

Supplement: Supplementary file 3 [file Image1.pdf]
